# Knotty-Centrality: Finding the Connective Core of a Complex Network

**DOI:** 10.1371/journal.pone.0036579

**Published:** 2012-05-09

**Authors:** Murray Shanahan, Mark Wildie

**Affiliations:** Department of Computing, Imperial College London, London, United Kingdom; Indiana University, United States of America

## Abstract

A network measure called knotty-centrality is defined that quantifies the extent to which a given subset of a graph’s nodes constitutes a densely intra-connected topologically central connective core. Using this measure, the knotty centre of a network is defined as a sub-graph with maximal knotty-centrality. A heuristic algorithm for finding subsets of a network with high knotty-centrality is presented, and this is applied to previously published brain structural connectivity data for the cat and the human, as well as to a number of other networks. The cognitive implications of possessing a connective core with high knotty-centrality are briefly discussed.

## Introduction

The mathematical theory of complex networks has developed a variety of measures that have found application in contemporary science, such as the small-world index, modularity, betweenness centrality, assortativity, and so on [1]. One such statistic is the *rich-club coefficient*, which can be used to capture the extent to which a network’s most highly connected nodes are densely connected among themselves [2]. The *rich-club coefficient* for degree *k* is defined as
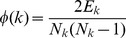
(1)where *E_k_* is the number of edges between nodes of degree greater than *k*, and *N_k_* is the number of such nodes [3]. If, for a given network, 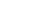 is unexpectedly low for low *k* and high for high *k*, then the network has a *rich club* of nodes that is densely connected to itself and “owns” a lot of the connectivity. An alternative degree-based assessment of network structure is provided by *k-core decomposition* [4], which involves the recursive removal of nodes below a given degree *k* until all remaining nodes in the network are of degree *k* or above. Incrementing *k* until the network is fully eroded yields a nested series of increasingly central *k*-cores.

**Figure 1:**
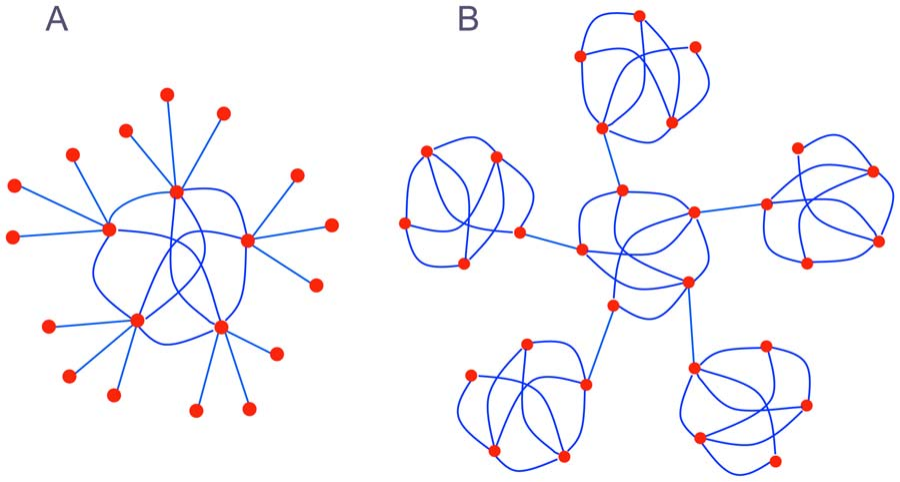
Example networks. (A) A network with a rich club. The set of nodes in the centre have high degree and are densely intra-connected. (B) A modular network with a knotty centre, but without a rich club. The set of nodes in the centre have high betweenness centrality, but their degree is no higher than the more peripheral nodes.

The present paper introduces a measure called *knotty-centrality* that attempts to capture a related concept, namely the extent to which the network possesses a densely intra-connected and topologically central core. To see that neither the rich-club coefficient nor *k*-core decomposition is always sufficient for this task, consider the two networks in Figure 1. On the left, we have a network in which the central five nodes form a rich club. They each have high degree, and they are densely connected to each other. By contrast, the fifteen nodes on the periphery have low degree and are connected only to members of the rich club. Similarly, the central five nodes are more resistant to *k*-core decomposition than the peripheral nodes. On the right we have a different kind of network. This network is highly modular, and each module has a connector hub through which it is connected to the rest of the network. Moreover, one of the modules is topologically central. All the other modules are connected to this central module, and none of them is connected to any other module. So every path between nodes in different peripheral modules passes through the central module. However, this central module does not constitute a rich club, nor is it resistant to *k*-core decomposition, because none of its nodes has unusually high degree. On the other hand, the nodes in this module do have unusually high betweenness centrality, and it is on this account that they can be picked out as the network’s “knotty centre”.

In this paper we formally define the concept of a “knotty centre” and apply it to a number of standard network models and real-world networks. We first present an algorithm for finding subsets of nodes within a directed graph that display high knotty-centrality. We then demonstrate that this measure captures an aspect of network structure that eludes previous measures by applying the algorithm to a set of randomly generated networks, encompassing several widely used topologies. We go on to apply the measure to a number of real-world networks, including the structural connectomes of the cat brain and human brain. We conclude with a brief discussion of the usefulness of knotty-centrality for understanding the neurological underpinnings of cognition.

## Methods

### Defining Knotty-Centrality

Consider a directed graph *G* with *N* nodes. The *knotty-centrality* of a (non-empty, non-singleton) subset *S* of the nodes in *G* is given by
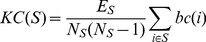
(2)where *E_S_* is the number of edges between nodes in *S*, and *N_S_* is the number of nodes in *S*. *bc*(*i*) is the betweenness centrality of node *i* normalised with respect to the whole graph, such that
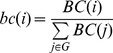
(3)where BC(i) is the (directed) betweenness centrality of node i as defined by Kintali [5]. Knotty-centrality ranges from 0 to 1. It is 0 if none of the nodes in S is adjacent 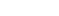 It is 1 if S is a clique and 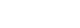 If G is a clique then 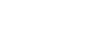 and 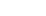 is undefined. The measure can be applied to either weighted or unweighted graphs by substituting weighted or unweighted variants of betweenness centrality [6] into Equation 3.

We can also weight knotty-centrality so that it favours small sub-graphs by taking account of the proportion of nodes excluded from *S*. The *compact knotty-centrality* of *S* is given by
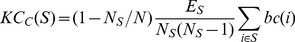
(4)


**Figure 2:**
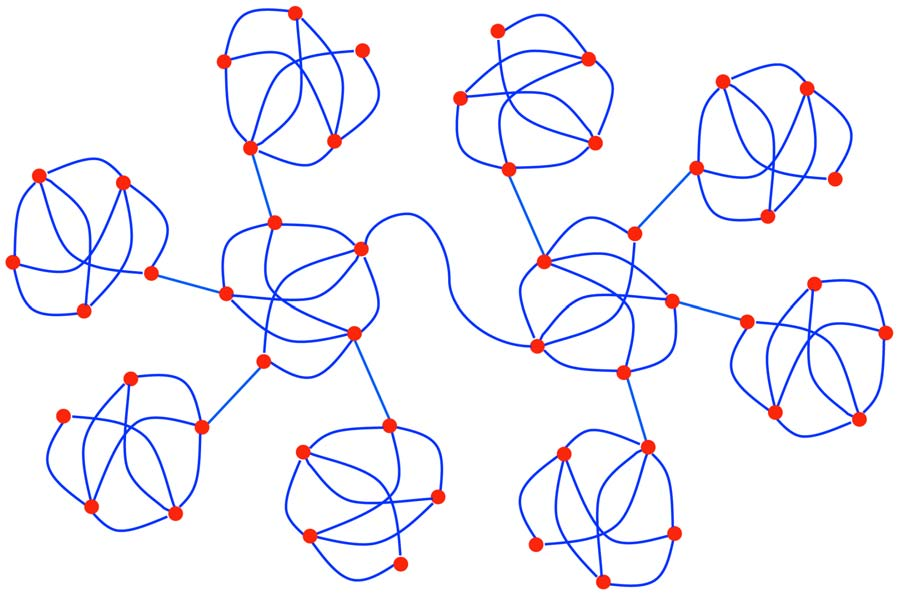
A network with two knotty centres.

A subset *S*_1_ of *G*’s nodes is a *knotty centre* of *G* if there does not exist a distinct subset *S*_2_ such that *KC*(*S*_2_) > *KC*(*S*_1_). There may be more than one knotty centre for a given graph, if they have equal knotty-centrality (Figure 2), but typically we will be interested in graphs that have a unique knotty centre. The *knotty-centredness* 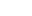 of the whole graph *G* is the knotty-centrality of its knotty centre(s). The definitions of a *compact knotty centre* and the *compact knotty-centredness* 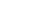 are analogous, but use *KC_C_* in place of *KC*.

To facilitate the comparison of graphs with different numbers of nodes and edges, we can define the *knotty-centre index* of a graph *G* as
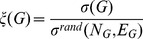
(5)where 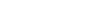 is the expected knotty-centredness of a random graph with the same number of nodes 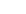 edges 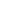 and the same degree sequence as *G*. If 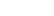 then *G* has a knotty centre, and the higher 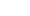 is the more pronounced that knotty centre is. Again, we obtain the compact knotty-centre index 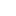 by substituting *KC_C_* for *KC*. Although normalisation by a random graph of the same degree sequence has been used to compare networks in several previous studies, we note that this can produce spurious results if the properties under study scale differently in the randomised version of the network [7].

### Computing Knotty-Centrality

There is no efficient naïve algorithm for finding the knotty centre of any given graph *G*. Obviously we could calculate the knotty-centrality of all 2*^N^* subsets of *G* and pick the one with the maximum value. But this is hopelessly inefficient for a non-trivial graph. An alternative is to exhaustively search all subsets of *G* whose members fall in the top *M* nodes for betweenness centrality, and then use gradient ascent (Figure S1). The exhaustive search phase is then O(2*^M^*), which is manageable if *M* is kept small.

As it stands the algorithm is non-deterministic. Any two nodes *i*, *j* that have equal betweenness centrality, and are connected by the same number of edges to the sub-graph *S*, will result in the same value 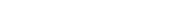 To render it deterministic let’s suppose that the nodes are numbered, and that the node with the highest number is chosen when there is a choice. Given that the algorithm employs gradient ascent, there is no guarantee of finding the optimal solution in the presence local maxima, where for some *S*_1_, *S*_2_ and *S*_3_

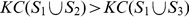
(6)and



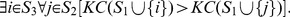
(7)A standard method of avoiding local maxima is to repeat the algorithm with some randomisation of initial conditions, such as the search order of vertices *V*. For the example brain connectivity matrices presented in this paper repetition with randomised *V* produced no improvement in 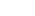 Substituting *KC_C_* for *KC* yields an analogous algorithm for approximating the compact knotty centre and estimating 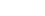 In what follows, deterministic versions of each algorithm with *M* = 10 will be assumed.

The basic algorithm of Figure S1 can be improved in two straightforward ways. First, the exhaustive search phase can be iterated. Having found *S*, the best subset of *G* among the top *M* nodes for betweenness centrality, a further exhaustive search can be carried out for the best extension of *S* that adds only nodes from the top *M* nodes in *G* not already included in *S*. This process can be repeated until *KC*(*S*) stops increasing, and then followed by a gradient ascent phase to catch any remaining nodes that might further increase *KC* despite their low-ranking betweenness centrality.

A second improvement can be made by using a different ranking for the nodes. In order to favour nodes that are connected to other nodes with high betweenness centrality, the “indirect” betweenness centrality of each node can be calculated. The *indirect betweenness centrality BC*′(*i*) of a node *i* is defined as
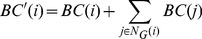
(8)where *N_G_*(*i*) is the set of nodes in *G* that are connected to *i* in either direction. The algorithm (Figure S2) that results when both improvements are incorporated was implemented in Matlab (see supporting information, Text S1), using a library function from the Brain Connectivity Toolbox to compute betweenness centrality [8]. While computationally expensive, the proposed algorithm proved sufficient for analysing real-world networks of the order of 5000 nodes and 10000 edges. In the case of larger graphs it may prove beneficial to increase the value *M* and hence size of the initial exhaustive search phase. Further investigation into efficient means of computing the measure is required for the analysis of larger networks.

## Results

To see how knotty-centrality works in practise, we first apply the measure to a number of well-known network models. To facilitate comparison with the rich-club coefficient, we define the *rich-club index* of a graph *G* in a similar manner to the knotty-centre index:
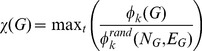
(9)where 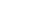 is the rich-club coefficient of graph *G* for degree *k*, and 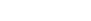 is the expected rich-club coefficient for degree *k* of a random graph with same number of nodes 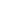 edges 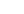 and the same degree sequence as *G*. 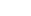 is then the maximum ratio for any degree *k* resulting in a rich club of size above threshold *t*.

**Figure 3:**
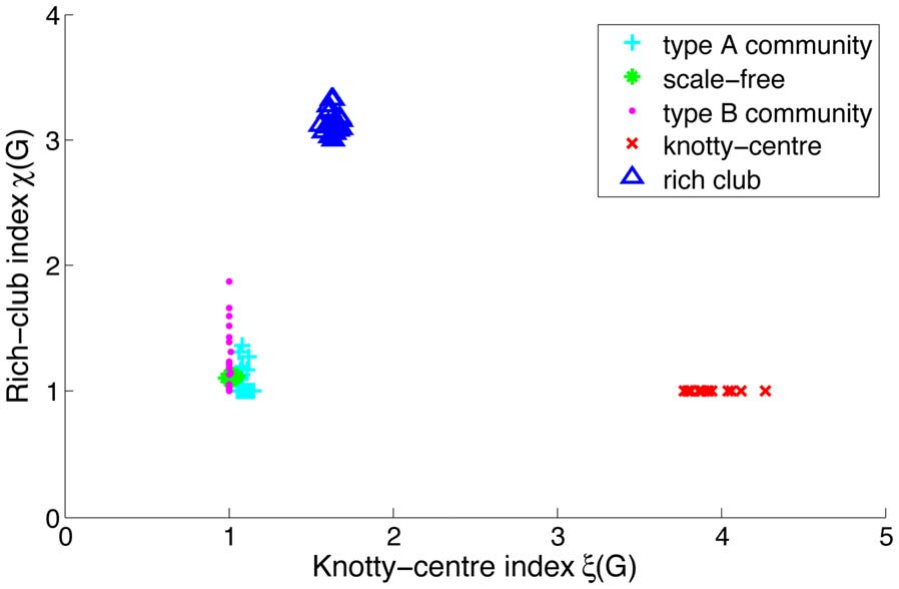
Knotty-centre index vs. rich-club index for three reference network models (type A and type B community-structured and scale-free). Two additional models are included with a central core network of either high degree (rich-club) or high betweenness centrality (knotty-centre).

In Figure 3 we compare rich-club and knotty-centre indices for scale-free and two types of community-structured networks. The dataset for each topology contains 20 randomly generated directed networks of 256 nodes each. For each network, we display 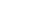 against 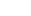 for rich-club threshold 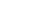 For generation of directed scale-free networks we used the algorithm described in [9] with parameters 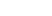 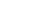 and 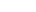 To generate the first type of community structured network (type A), we used probabilistic re-wiring [10] with eight modules of 32 nodes each. Each node was randomly connected to 20 nodes within the same module, and each edge re-wired to an external module with probability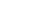 Community-structured networks of the second type (type B) were generated according to the description in [11] again with eight communities of 32 nodes, and with probability of internal wiring 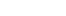 and of external wiring 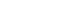 For each network a set of 100 random surrogate networks of the same degree sequence was generated using a library function from the Brain Connectivity Toolbox [8] and used to calculate both rich-club and knotty-centre indices.

We include two additional topologies generated specifically to maximise both measures. In the knotty-centre case, eight modules of 32 nodes were connected internally in a similar manner to the modular small-world network. Instead of randomly re-wiring edges between modules however, a single module was selected as the centre of the network and a pair of random nodes connected in either direction between the centre module and each non-centre module. The probability *p_im_* of connecting to any node *i* in module *m* of graph *G* was then adjusted at each step during generation of internal module connectivity, such that
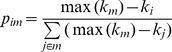
(10)where 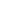 is the degree of node *i,* and 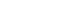 is the maximum degree for all nodes in module *m*. Given an equal number of internal connections in each module, this results in a set of topologically central nodes of high betweenness centrality with maximum difference in incoming degree of at most one for all nodes in *G*. In the rich club case, each node was first connected to sixteen others randomly selected from the entire network. A subset of nodes was then selected to form a rich club, and additional edges added from each of these nodes to four other randomly selected rich club nodes, resulting in an intra-connected high-degree sub network. For all networks described above, multiple edges in the same direction between the same source and destination nodes and connections between a node and itself were disallowed.

Results are shown in Figure 3. For the standard network models, neither knotty-centre index (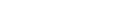 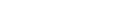 and 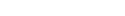 for type A community-structured, type B community-structured, and scale-free networks respectively) nor rich-club index (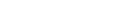 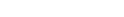 and 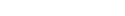 respectively) indicate that the generated networks exhibit a more pronounced rich club or knotty centre than randomised equivalents, although both community-structured models exhibit a large range of rich-club values, within [1.0, 1.8]. The two topologies explicitly generated with a rich club or knotty centre both display high values of a single index (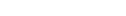 and 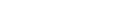 for rich-club and knotty-centred networks and indices respectively). This indicates that the rich-club and knotty-centre measures capture different aspects of network topology, neither of which is consistently displayed in the networks generated by current standard models. It is worth noting that rich-club networks also exhibit a knotty centre 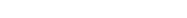
_ while networks generated with high knotty-centrality do not exhibit a rich club 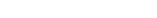._

We now briefly consider the stability of the knotty-centre index when intra-module connections are added between the outer modules of a network of the form shown in Figure 1B. A slight modification is made to the randomly generated knotty-centre network described above, by adding a single edge in either direction between each adjacent module outside of the centre module. This effectively short-circuits the shortest path between modules around the rim of the network. For each topology, with and without connections between modules outside the central core, we generated 100 random networks. Without intra-module connections, networks exhibited average knotty-centre index of 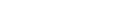 and size of the knotty centre of 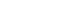 nodes. With intra-module connections networks exhibited average knotty-centre index of 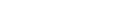 and size of 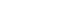 nodes. The change to the index value and size of the knotty centre is in-line with the loss of topological centrality of the core module resulting from the additional connections.

We turn next to real-world data sets, choosing two previously used networks that exhibit a knotty centre. We consider first the power grid of the Western United States used as an example of a small-world network in [12]. The network consists of 4941 nodes and 6594 edges with few connections between nodes of high degree. Calculation of the rich-club index against 100 randomized versions of the same network results in a value of 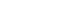 for degree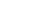 indicating a rich club comprising 26 nodes of degree 20 or greater. Computing the knotty-centre index identifies a subset of nodes (2529, 2544, 2607, and 2613) with high knotty-centrality compared to equivalent random networks 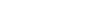 with no overlap between the rich club and knotty centre. Each of these nodes exhibits low degree (6, 6, 7, and 4 respectively, where the maximum degree in the network is 19) but is highly central, with the combined betweenness centrality of the four nodes representing approximately 4.7% of the network total. Together they form a highly connected network with four of six possible edges present.

The second real-world dataset we consider is the co-authorship network of scientists working on network theory and experiment described in [13]. We consider a binarised (unweighted) version of the network containing 1589 nodes and 2742 edges. Comparison against 100 randomised versions of the same network yielded an estimate of 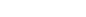 and 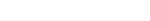 indicating a rich club of index 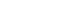 comprising 27 nodes of degree 36 or greater. This network possesses a knotty centre (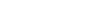 and 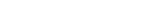 giving 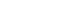) comprising a network of eight nodes (ids 79, 151, 152, 226, 282, 302, 517, and 518). The degrees of these nodes fall in the range [6], [24] compared to a network maximum of 34. They are of high centrality (combined betweeness centrality 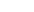 of network total), and are highly connected, with 14 of 28 possible edges present. The rich club and knotty centre overlap by a single node (node 79, who is M.E.J. Newman, the paper’s author). It is worth noting that a number of subsets of any network are likely to exist with near-optimal knotty-centrality [14], and in real-world examples it may be instructive to build a profile of larger groups of nodes with high knotty-centrality.

**Figure 4:**
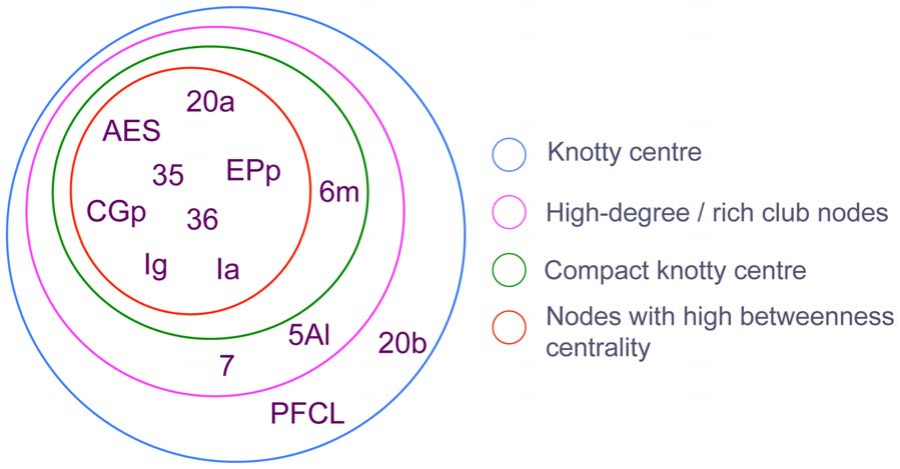
The knotty centre of cat cortex and its relationship to other topologically significant subsets of nodes. There is good agreement in this case between rich club membership, high betweenness centrality, and knotty-centrality.

We next apply the measure to a number of brain networks. The first is a connectivity matrix *G_cat_* for the cortex of the cat described in [15]. This was collated from a large number of tracer studies of adult cat cortical and thalamic connectivity, and represents a single hemisphere containing approximately 1500 connections parcellated into 95 anatomical regions. It was analysed from a graph-theoretic standpoint by Sporns, *et al*. [16], and further studied by Zamora-López, *et al*. [17] from a perspective close to that of the present paper. Both binarised (unweighted) and weighted versions of the 52 cortical regions of the matrix were used for the present study. The matrix has 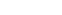 nodes and 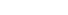 edges. By generating 100 random directed networks with *N_cat_* nodes, *E_cat_* edges, and the same degree sequence as *G_cat_*, an estimate of 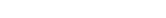 was obtained for the unweighed matrix. For *G_cat_* itself an estimate 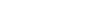 was obtained, yielding knotty-centre index of 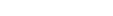 The membership of the computed knotty centre, using the nomenclature of Scannell, *et al*. (1999) [15], was {20a, 20b, 7, AES, EPp, 6 m, 5 Al, PFCL, Ia, Ig, CGp, 35, 36} (Figure 4). This includes all eleven nodes having degree greater than one standard deviation above the mean, a set that is also identified as both a rich club and a dynamic core by Zamora-López, but also includes areas 20 b and PFCL, and includes all eight of the nodes having betweenness centrality greater than one standard deviation above the mean, as well as areas 7, 6 m, 5 A1, 20 b, and PFCL.

Similarly, estimates of 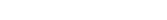 and 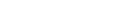 were obtained, yielding a compact knotty-centre index of 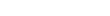. The membership of the computed compact knotty centre was {20a, AES, EPp, 6 m, Ia, Ig, CGp, 35, 36} (Figure 4). This comprises nine out of the eleven high-degree/rich-club nodes, excluding only areas 7 and 5 A1, and includes all eight of the nodes having betweenness centrality greater than one standard deviation above the mean, as well as area 6 m. The weighted matrix resulted in similar values of 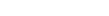 and 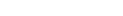 To summarise, the knotty centre of the feline brain has a large overlap with the subset of nodes that have previously been identified as topologically significant using other measures. Moreover, the knotty-centre index captures in a single measure the considerable extent to which this distinguished set of nodes stands out as a topological nexus over and above any subset of nodes in a comparable random network.

**Figure 5:**
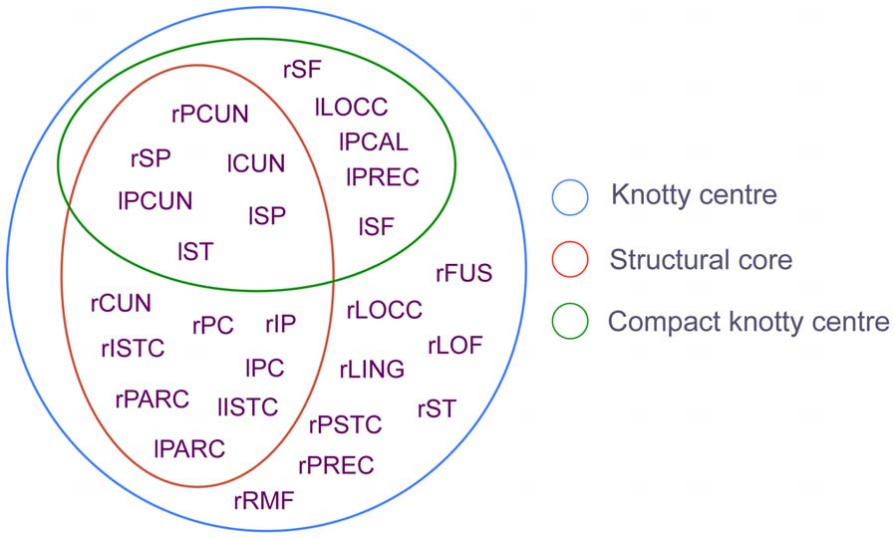
The knotty centre of human cortex and its relationship to the structural core as defined by Hagmann, *et al*. (2008) [**18**]. The compact knotty centre has a large overlap with the structural core, but excludes central medial areas and includes additional superior frontal areas.

To further assess its utility, the measure was applied to a second brain network, namely the structural connectivity matrix *G_hum_* derived from diffusion spectrum imaging of five subjects by Hagmann, *et al*. [18] and subjected to a graph-theoretic analysis by the same authors. The matrix contains 66 cortical regions partitioned according to standard anatomical landmarks. Connectivity is based on the density and length of white matter fibre tracts connecting each region. As with the cat matrix, we consider both binarised (unweighted) and weighted versions of the human matrix in the present study, for which we have 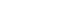 nodes and 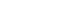 edges. 100 random directed networks with *N_hum_* nodes, *E_hum_* edges, and the same degree sequence as *G_hum_* were generated in each case. For the unweighed matrix this yielded an estimate of 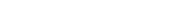 The knotty- centredness of *G_hum_* was estimated as 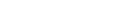 yielding an estimated knotty-centre index of 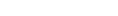 Although the computed knotty centre includes all members of the “structural core” identified by Hagmann, *et al*. [18], it comprises over 40% of the whole network (Figure 5).

Following the same procedure with the compact version of the measure yielded 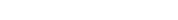 and 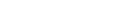 giving 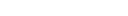 The computed compact knotty centre comprises 11 nodes (Figure 5). Of these, six are shared with the Hagmann structural core, including the precuneus and superior parietal areas in both hemispheres. However, it excludes eight regions that are contained in the Hagmann structural core, and contains five that are not. The five extra regions are predominantly in the left hemisphere, but include superior frontal areas in both hemispheres. The weighted matrix resulted in values 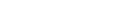 and 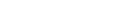


It will be noted that the two knotty-centre indices for the human matrix are exceeded by the corresponding indices for the cat matrix, which may seem counter-intuitive (even to a cat-lover). Given that the cat matrix represents a single hemisphere, the analysis was also repeated for each hemisphere of the human connectivity matrix independently, returning values in both cases lower than that of the combined matrix (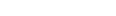 and 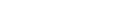 for the left hemisphere and 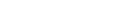 and 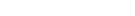 for the right). It should be remembered that the human and cat matrices were produced using different methods, and as noted previously [7], comparison of the graph theoretic properties of networks of different size and connectivity is not straightforward. A legitimate cross-species comparison would require directly comparable matrices, and would need to treat variations in connection strength more carefully.

## Discussion

The present results suggest that the concept of knotty-centrality could make a useful addition to the network analysis toolbox. Although the ideal knotty centre of a graph is hard to compute, the approximations found by the heuristic algorithm proposed here correspond well with previous more *ad hoc* attempts to find topologically significant subsets of brain networks. The measure requires further work, however. It is unclear, for example, whether compact knotty-centrality is more or less informative than the non-compact version, or whether in fact both measures should be retained. The issue of multiple knotty centres, which may be relevant to split-brain patients for example, remains to be studied. Moreover, in the present work, no distinction is made between nodes that feature in sets with less than but close to maximal knotty-centrality and nodes that feature only in sets with low knotty-centrality. So it may be desirable, rather than searching for an ideal knotty centre, to characterise families of (possibly overlapping) sets of nodes with high knotty-centrality. Finally, an investigation of the dynamical implications of knotty-centrality would be beneficial, either using empirical or synthetic data. In the latter case, algorithms for generating graphs with varying degrees of knotty-centrality would be needed.

As a measure of topological centrality, knotty-centrality stands apart from degree-based measures of network structure such as the rich-club coefficient and *k*-core decomposition. Nodes forming a rich club by definition have higher degree than the remaining nodes in the network and high connectivity between the rich club members. Similarly *k*-core decomposition identifies sets of highly connected nodes, and *s*-core decomposition [18] sets of strongly connected nodes in a weighted network. By contrast, there is no constraint on the degree or strength of connectivity of nodes forming a knotty centre. The randomly generated knotty-centred networks considered in this paper explicitly maintain uniform degree of nodes and equal weight of edges while generating a topologically central core module. All of these measures can be considered complementary views into network topology.

Although it may have application in other domains, the concept of knotty-centrality is intended as an aid to identifying a connective core in the brains of humans and other animals. Recent evidence supports the notion of a topologically and spatially central core network linking all areas of the brain and supporting efficient global communication [19]. The possession of a (single) connective core potentially constrains the way information flows around the brain in a way that a) promotes the generation of integrated brain states [20], and b) facilitates serial processing [21], providing the flexibility to cope with an arbitrarily large number of complex tasks [22]. It is hypothesised further that a central core acts simultaneously as an arena for competition and a locus of broadcast, mediating the interactions of numerous otherwise segregated elements and allowing the brain to enter a state in which their activity is coherently integrated [23], [24]. Perhaps, using the formal concept of knotty-centrality, it will be possible to identify networks of brain regions that fulfil these vital cognitive roles in a variety of species.

## Supporting Information

Figure S1 An algorithm for computing a subset of nodes with high knotty-centrality.
(DOCX)

Figure S2 An improved algorithm for computing a subset of nodes with high knotty-centrality.
(DOCX)

Text S1 Matlab code for finding the knotty centre of a graph.
(DOC)
