# Supplementary material for: Knotty-Centrality: Finding the Connective Core of a Complex Network
Source: PLoS One. 2012 May 9;7(5):e36579. doi: 10.1371/journal.pone.0036579 (PMC3348887; doi:10.1371/journal.pone.0036579)
Supplement: Figure S1 — An algorithm for computing a subset of nodes with high knotty-centrality. (DOCX) [file pone.0036579.s001.docx]

V := list of vertices of G

sort V by betweenness centrality

let V1 be the first M vertices in V

find S⊂V1 such that KC(S) is maximal

V := V-S

done := false

**while** V ≠ {} and not done

find some i∈V such that KC(S∪{i}) is maximal

**if** KC(S∪{i})>KC(S)

S := S∪{i}

V := V-{i}

**else** done = true

**end**
