# Supplementary material for: Knotty-Centrality: Finding the Connective Core of a Complex Network
Source: PLoS One. 2012 May 9;7(5):e36579. doi: 10.1371/journal.pone.0036579 (PMC3348887; doi:10.1371/journal.pone.0036579)
Supplement: Figure S2 — An improved algorithm for computing a subset of nodes with high knotty-centrality. (DOCX) [file pone.0036579.s002.docx]

V := list of vertices of G

sort V by indirect betweenness centrality

S := {}

done := false

**while** not done

M := min(M,length(V))

let V1 be the first M vertices in V

find S2⊂V1 such that KC(S∪S2) is maximal

**if** S2 ≠ {}

S := S∪S2

V := V-S2

**else** done := true

**end**

done := false

**while** V ≠ {} and not done

find some i∈V such that KC(S∪{i}) is maximal

**if** KC(S∪{i})>KC(S)

S := S∪{i}

V := V-{i}

**else** done = true

**end**
