# Supplementary material for: Knotty-Centrality: Finding the Connective Core of a Complex Network
Source: PLoS One. 2012 May 9;7(5):e36579. doi: 10.1371/journal.pone.0036579 (PMC3348887; doi:10.1371/journal.pone.0036579)
Supplement: Text S1 — Matlab code for finding the knotty centre of a graph. (DOC) [file pone.0036579.s003.doc]

function [nodes kc] = FindKnottyCentre(CIJ,compact)

% Attempts to find the sub-graph of CIJ with the highest value for

% knotty-centrality. Carries out a series of exhaustive searches on

% subsets of the nodes ranked by "indirect" betweenness centrality, then

% carries out a phase of hill-climbing to see whether the sub-graph can

% be improved by adding further nodes. Uses the Brain Connectivity

% Toolbox (Rubinov & Sporns, 2010) for betweenness centrality

%

% nodes = the sub-graph found

% kc = its knotty-centredness

% compact = 1 if compact knotty-centrality to be used, 0 otherwise

%

% Written by Murray Shanahan, October 2011

N = length(CIJ);

CIJ = (CIJ > 0).*1; % binarise matrix - all non-zero weights become 1s

% Exhastive search phase

Exh = 10; % number of nodes for exhaustive search (2^Exh combinations)

Exh = min(Exh,N);

BC = betweenness_bin(CIJ); % betweenness centralities (Rubinov & Sporns)

BC = BC/sum(BC); % normalise wrt total betweenness centrality

% Calculate indirect betweenness centrality

BC2 = zeros(1,N);

for i = 1:N

BC2(i) = BC(i)+(sum(CIJ(i,:).*BC(i)))+(sum(CIJ(:,i)'.*BC(i)));

end

[~, IxBC] = sort(BC2,'descend'); % rank nodes

nodes = [];

improving = 1;

while improving

L = length(nodes);

nodes_left = IxBC;

nodes_left = nodes_left(~ismember(nodes_left,nodes));

choices = nodes_left(1:min(Exh,end));

[nodes kc] = BestPerm(nodes,choices,CIJ,compact,BC);

improving = length(nodes) > L;

end

% Hill climbing phase

nodes_left = 1:N;

nodes_left = nodes_left(~ismember(nodes_left,nodes));

improving = 1;

while improving && ~isempty(nodes_left)

best_kc = 0;

for i = 1:length(nodes_left)

node = nodes_left(i);

nodes2 = [nodes, node];

kc2 = KnottyCentrality(CIJ,nodes2,compact,BC);

if kc2 > best_kc

best_kc = kc2;

best_node = node;

end

end

if best_kc > kc

kc = best_kc;

nodes = [nodes, best_node];

nodes_left = nodes_left(nodes_left ~= best_node);

else

improving = 0;

end

end

end

function [nodes,kc] = BestPerm(given,choices,CIJ,compact,BC)

% Carries out exhaustive search to find a permutation of nodes in

% "choices" that when added to the nodes in "given" yields the highest

% value of knotty-centrality

if ~isempty(choices)

choices2 = choices(2:end);

new = choices(1);

[nodes1,kc1] = BestPerm([given, new],choices2,CIJ,compact,BC);

[nodes2,kc2] = BestPerm(given,choices2,CIJ,compact,BC);

if kc1 > kc2

nodes = nodes1;

kc = kc1;

else

nodes = nodes2;

kc = kc2;

end

else

nodes = given;

kc = KnottyCentrality(CIJ,nodes,compact,BC);

end

end

function kc = KnottyCentrality(CIJ,nodes,compact,BC)

% Returns knotty-centrality of the subgraph of CIJ comprising only

% "nodes" and the associated connections

if length(nodes) < 3

kc = 0;

else

CIJ = (CIJ > 0).*1; % binarise matrix

N = length(CIJ); % nodes in overall graph

M = length(nodes); % nodes in subgraph

BCtot = sum(BC(nodes));

p = ((N-M)/N); % proportion of nodes not in subgraph

RC = sum(sum(CIJ(nodes,nodes)))/(M*(M-1)); % density of subgraph

if compact

kc = p*BCtot*RC; % compact knotty-centrality

else

kc = BCtot*RC; % knotty-centrality

end

end

end
